# Supplementary material for: Impact of COVID-19 Pandemic on Children Visiting Emergency Department for Mental Illness: A Multicenter Database Analysis from Korea
Source: Children (Basel). 2022 Aug 11;9(8):1208. doi: 10.3390/children9081208 (PMC9406735; doi:10.3390/children9081208)
Supplement: Supplementary file 1 [file children-09-01208-s001.zip › children-1842108-supplementary.pdf]

Supplemental Table S1. Subgroups of mental illness by ICD-10 code.

Diagnosis	ICD code
Anxiety disorders	F40, F41, F42, F48.8–F48.9
Mood disorders	F06.3, F30–F34
Somatoform disorders	F45
Developmental disorders	F64.2, F70–79, F80–89, F84, F93–F95, F98
Schizophrenia and other psychotic disorders	F06.0–F06.2, F20–F29
Alcohol use disorder	F10, F55
Intentional self-harm	X60–X84
Medicine use disorder	F11–F19, T36–T50
Miscellaneous	F02.8, F03.9, F04, F05, F07, F09, F43, F44, F48.1–F48.2, F50–F59, F60–F69, F90, F91, F99
